# Chitosan-miRNA functionalized microporous titanium oxide surfaces via a layer-by-layer approach with a sustained release profile for enhanced osteogenic activity

**DOI:** 10.1186/s12951-020-00674-7

**Published:** 2020-09-09

**Authors:** Kaimin Wu, Mengyuan Liu, Nan Li, Li Zhang, Fanhui Meng, Lingzhou Zhao, Min Liu, Yumei Zhang

**Affiliations:** 1 Department of Stomatology, Navy 971st Hospital, No. 22 Minjiang Road, Qingdao, 266071 China; 2 Oral Research Center, Qingdao Municipal Hospital, Qingdao, 266071 China; 3 Third Department of Cadre’s Ward, Navy 971st Hospital, Qingdao, 266071 China; 4 State Key Laboratory of Military Stomatology & National Clinical Research Center for Oral Diseases & Shaanxi Key Laboratory of Stomatology, Department of Prosthodontics, School of Stomatology, The Fourth Military Medical University, No. 145 West Changle Road, Xi’an, 710032 China; 5 State Key Laboratory of Military Stomatology & National Clinical Research Center for Oral Diseases & Shaanxi Engineering Research Center for Dental Materials and Advanced Manufacture, Department of Periodontology and Oral Medicine, School of Stomatology, The Fourth Military Medical University, No. 145 West Changle Road, Xi’an, 710032 China

**Keywords:** microRNAs, Sustained release, Layer-by-layer, Microarc oxidation, Mesenchymal stem cells, Titanium implants

## Abstract

**Background:**

The biofunctionalization of titanium implants for high osteogenic ability is a promising approach for the development of advanced implants to promote osseointegration, especially in compromised bone conditions. In this study, polyelectrolyte multilayers (PEMs) were fabricated using the layer-by-layer approach with a chitosan-miRNA (CS-miRNA) complex and sodium hyaluronate (HA) as the positively and negatively charged polyelectrolytes on microarc-oxidized (MAO) Ti surfaces via silane-glutaraldehyde coupling.

**Methods:**

Dynamic contact angle and scanning electron microscopy measurements were conducted to monitor the layer accumulation. RiboGreen was used to quantify the miRNA loading and release profile in phosphate-buffered saline. The in vitro transfection efficiency and the cytotoxicity were investigated after seeding mesenchymal stem cells (MSCs) on the CS-antimiR-138/HA PEM-functionalized microporous Ti surface. The in vitro osteogenic differentiation of the MSCs and the in vivo osseointegration were also evaluated.

**Results:**

The surface wettability alternately changed during the formation of PEMs. The CS-miRNA nanoparticles were distributed evenly across the MAO surface. The miRNA loading increased with increasing bilayer number. More importantly, a sustained miRNA release was obtained over a timeframe of approximately 2 weeks. In vitro transfection revealed that the CS-antimiR-138 nanoparticles were taken up efficiently by the cells and caused significant knockdown of miR-138 without showing significant cytotoxicity. The CS-antimiR-138/HA PEM surface enhanced the osteogenic differentiation of MSCs in terms of enhanced alkaline phosphatase, collagen production and extracellular matrix mineralization. Substantially enhanced in vivo osseointegration was observed in the rat model.

**Conclusions:**

The findings demonstrated that the novel CS-antimiR-138/HA PEM-functionalized microporous Ti implant exhibited sustained release of CS-antimiR-138, and notably enhanced the in vitro osteogenic differentiation of MSCs and in vivo osseointegration. This novel miRNA-functionalized Ti implant may be used in the clinical setting to allow for more effective and robust osseointegration.
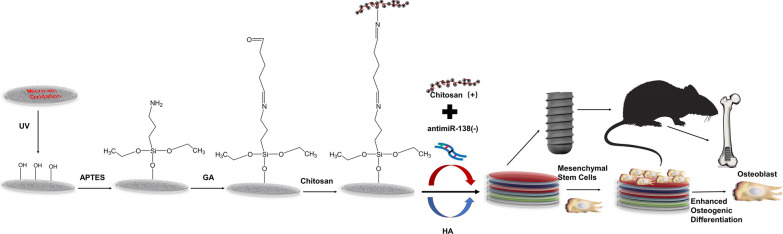

## Introduction

Titanium (Ti) implants are widely applied as orthopaedic and dental implants. Although these implants have been successfully employed in the past, further development is required to meet the clinical needs for faster and tighter osseointegration [1, 2]. Conventional implant modification techniques, such as alteration of surface properties (e.g., suitable micro-, submicro- and nanoscale topographies and wettability [3–6]) and loading and delivery of inorganic elements [7, 8] are suggested to have limited effects on osteogenic activity enhancement [9]. Biofunctionalization of Ti implants with potent biochemical cues, such as extracellular matrix (ECM) proteins [10], peptides [11, 12], growth factors [13, 14] and nucleotides [15, 16], may result in more predictable osseointegration, especially in compromised bone conditions.

MicroRNAs (miRNAs) are endogenous small non-coding RNAs 19–25 nucleotides in length that play an important role in a variety of diverse and fundamental biological processes, such as cell development, proliferation, differentiation, apoptosis and signal transduction. It is believed that miRNAs mimic the natural differentiation pathway and delicately control multiple genes, constituting a much healthier stimulator of stem cell differentiation [17]. Recently, reports on the regulatory effect of miRNAs on osteogenesis have continued to expand [18–21], among which miR-138 is reported to be a negative regulator of the osteogenic differentiation of mesenchymal stem cells (MSCs). AntimiR-138 inhibition of endogenous miR-138 levels was reported to enhance bone formation in vivo [18]. In our previous proof-of-concept studies, antimiR-138 lipoplexes were introduced to either the cell culture plate or the Ti surface to enhance the osteogenic differentiation of the MSCs cultured on these surfaces [22]. Before possible clinical application, it is important to use a more biocompatible carrier instead of liposomes and to control the loading and delivery kinetics of antimiR-138 to achieve a sustainable effect.

As a natural degradable cationic polymer, chitosan (CS), with good stability and cytocompatibility, could enhance drug internalization [23, 24] and has already been used for the delivery of DNA and RNA [25–28]. Via electrostatic interactions, CS can bind miRNAs to form relatively stable CS-miRNA nanoplexes (NPs) with a positive charge. Positively and negatively charged polyelectrolytes can be alternately adsorbed via electrostatic adsorption to fabricate a polyelectrolyte multilayer (PEM) film; this is referred to as layer-by-layer (LbL) technology [29, 30]. PEMs with controlled loading and release profiles has been experimentally used to deliver growth factors [31, 32], DNA molecules [33, 34], RNA molecules [35, 36], etc.

It would be of special interest to use CS-antimiR-138 NPs and hyaluronic acid (HA, a commonly used polyanion in LbL) to PEM-functionalize Ti bone implants for sustainable antimiR-138 delivery and consequently augmented osseointegration. In addition, a more stable immobilization of the first layer is expected from chemical pretreatment of Ti with (3-aminopropyl) triethoxy silane (APTES) and glutaraldehyde (GA).

**Scheme 1:**
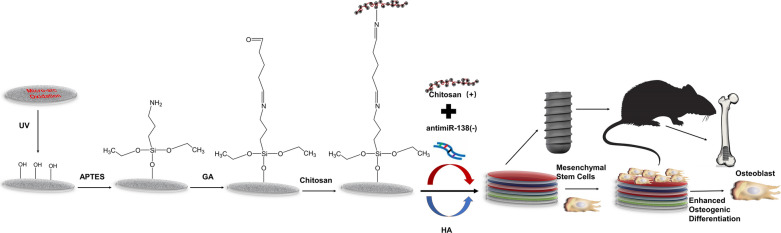
Schematic diagram summarizing the fabrication of the CS-antimiR-138/HA PEM-functionalized microporous Ti implant through LbL with enhanced osteogenic activity

In this study, we developed a novel CS-antimiR-138/HA PEM-functionalized microarc-oxidized (MAO) Ti implant by LbL and silane-glutaraldehyde coupling (Scheme 1). Following ultraviolet (UV) irradiation, a high number of surface hydroxyl groups was achieved by the facile and economical MAO treatment of Ti with high loading capacity [37]. The CS amine groups can form covalent bonds with the hydroxyl groups via silane-glutaraldehyde coupling of APTES and GA [38–40]. Furthermore, the osteogenic antimiR-138 was chosen for the PEM functionalization of the MAO Ti surface through LbL, and sustainable antimiR-138 delivery and enhanced osteogenic activity in vitro and in vivo was expected. We hope this work will provide beneficial information for loading miRNAs into other biomaterials.

## Materials and methods

### Materials

Commercially pure Ti discs (15 mm in diameter and 2 mm in thickness) and screw Ti rods (2.8 mm in diameter and 6 mm in length) were provided by the Northwest Institute for Nonferrous Metal Research, China. CS (molecular weight 100 kDa and deacetylation degree of 95%) was purchased from Jinke Co. Ltd (China). β-glycerophosphate disodium salt pentahydrate, calcium acetate monohydrate, APTES, GA, ascorbic acid, dexamethasone, Sirius Red, Alizarin Red and dimethyl sulfoxide (DMSO) were purchased from Sigma-Aldrich Company (USA). Solution RNase Erasol was bought from TIANDZ (China). α-minimal essential medium (α-MEM), fetal bovine serum (FBS), penicillin and streptomycin were obtained from Hyclone (USA). Cell count kit-8 (CCK-8), lactate dehydrogenase (LDH), 3,39-dioctadecyloxacarbocyanine perchlorate (DiO) and BCIP/NBT Alkaline Phosphatase Color Development Kit were provided by Beyotime (China). Phosphate buffered saline (PBS), 4′,6-diamidino-2-phenylindole (DAPI) were purchased from Invitrogen (USA). E.Z.N.A.™ Total RNA Kit I was obtained from OMEGA (USA). PrimeScriptTM RT reagent kit and SYBR Premix ExTM Taq II were purchased from TaKaRa (Japan). Male SD rats (2 weeks old) were obtained from the laboratory animal center of the Fourth Military Medical University. The antimiR-138 (CGGCCUGAUUCACAACACCAGCU) and negative control (CAGUACUUUUGUGUAGUACAA) were synthesized from Gene Pharma Co., Ltd (China).

### Fabrication of CS-antimiR-138/HA PEM-functionalized microporous Ti through LbL

Pure Ti discs were polished with 400- to 1500-grit waterproof abrasive paper and then ultrasonically cleaned in acetone, ethanol and distilled water for 10 min each. The Ti discs were then treated in an electrolyte containing β-glycerophosphate disodium salt pentahydrate (0.02 M) and calcium acetate monohydrate (0.2 M) at 400 V DC for 5 min. After ultrasonic cleaning and drying, the fabricated MAO Ti samples were sterilized for 30 min by UV irradiation.

CS and HA were dissolved in 0.2 M sodium acetate buffer (pH = 5.5) and deionized water (pH = 5.5), respectively, at a concentration of 1 mg/ml. Both CS and HA were sterilized using a 0.22 μm syringe filter and then treated with the Solution RNase Erasol Kit according to the manufacturer’s instructions. Afterwards, 100 μl antimiR-138 (20 mM in RNA-free water) was added to 1 ml final CS solution, quickly followed by magnetic stirring for 10 min and incubation for 30 min at room temperature. The N/P ratio (the molar ratio of chitosan amino groups to RNA phosphate groups) in our experiment was 60, calculated with a mass per phosphate of 325 Da for RNA and mass per charge of 163 Da for chitosan (95% deacetylation).

The CS-antimiR-138/HA PEM coating was then fabricated on MAO Ti surfaces via LbL. The dried MAO Ti discs were immersed in a 10% (v/v) solution of APTES in alcohol in sealed individual containers for 2 h on a shaker. Then, the MAO discs were placed in pure alcohol and sonicated for 30 min, which was repeated twice for a total sonication time of 90 min. Afterwards, the Ti discs were rinsed with deionized water twice to remove residual alcohol and then dried. To link GA to the MAO-APTES surface, the GA solution (2.5% (v/v) in deionized water) was poured over the MAO-APTES samples ensuring complete coverage of the metal coupon. The containers were then sealed for 1 h. Afterwards, the samples were rinsed thoroughly with deionized water 3 times. The CS dissolved in 0.2 M sodium acetate buffer (10 mg/ml, pH 5.5) was added to the Ti surface for 20 min. After washing with sodium acetate buffer, the Ti sample was alternatively dipped into the HA solution and CS-antimiR-138 solution for 10 min each. Every dipping step was followed by two washing steps with sodium acetate buffer to remove excess materials. The steps were repeated until the desired number of bilayers was obtained.

### Characterization of the CS-antimiR-138/HA PEM-functionalized microporous Ti

The surface morphology of the prepared Ti samples was observed by field-emission scanning electron microscopy (FE-SEM, Hitachi S-4800, Japan). To assess the miRNA loading, Cy3-labelled miRNAs (Gene Pharma, China) were used to fabricate the coating. The Ti samples were observed by a laser scanning confocal microscope with a layer by layer scanning approach with an interlayer thickness of 400 nm (Fluo View, Olympus FV1000, Japan) immediately after fabrication or after 7 days of incubation in α-MEM supplemented with 10% FBS. The three-dimensional images were reconstructed.

### Quantification of the loading and release of miRNA from CS-antimiR-138/HA PEM-functionalized microporous Ti

To measure the miRNA loading within each layer of the functionalized surface, droplets of 40 μl CS-miRNA nanoparticles were dropped discreetly in the experiment and incubated for 10 min. Then, the coated surface was washed twice with 40 μl sodium acetate buffer. Afterwards, the washing solution was carefully collected and quantitatively analysed by the RiboGreen assay according to the manufacturer’s instructions. Finally, the miRNA loading in each layer was calculated by subtracting the miRNA amount in the washing solution from the amount in the original 40 μl CS-miRNA nanoparticle suspension. The total miRNA loading in the PEMs was calculated as the sum of the miRNA amount in each layer.

The CS-antimiR-138/HA PEM-functionalized microporous Ti samples were incubated in 300 μl PBS (pH = 7.4) at 37 °C in 5% CO_2_ and 100% humidity for 14 days. At 8, 16, and 24 h and 2, 3, 4, 6, 8, 10, 12 and 14 days, the extracting solution was replaced with 300 μl of fresh PBS solution. The released miRNA in the collected PBS solution was quantified with the RiboGreen assay. As a reference, a standard curve of the miRNA concentration was determined with multiple dilutions of miRNA in PBS and PBS alone.

### Cell culture

Primary rat bone marrow MSCs were obtained from 2-week-old Sprague–Dawley rats. The cells were cultured in α-MEM supplemented with 10% FBS and 1% penicillin/streptomycin and incubated in a humidified atmosphere of 5% CO_2_ at 37 °C. Passages 2–4 were used in the experiments. The MAO surface and tissue culture plate served as controls. The medium was replaced twice every week.

### Transfection efficiency assay

Functionalized Ti samples made of Cy3-labelled miRNAs were used for this assay. MSCs of 2.5 × 10^4^/cm^2^ were inoculated on Ti samples placed in 24-well plates. After 48 h of culture, the transfected cells were harvested by treatment with trypsin, washed with PBS and fixed in 1% paraformaldehyde. To observe the internalization of the Cy3-labelled miRNAs into the cells, the cells were fixed with 4% paraformaldehyde and washed with PBS. The cell membrane was stained with DiO. The cell nucleus was stained with DAPI. The DiO, DAPI and Cy3 fluorescence signals were then observed by laser scanning confocal microscopy. To measure the miR-138 amount in the cells, the RNA in the cells was collected and reverse-transcribed using a PrimeScript RT reagent kit and a specific reverse transcription primer (Gene Pharma, China) according to the manufacturer’s recommendation. The miR-138 amount was quantified using a real-time polymerase chain reaction (real-time PCR) system (CFX96™, Bio-Rad, USA) with SYBR Premix Ex Taq™ II. U6 small nuclear RNA was used as an endogenous normalization control.

### Cell morphology

MSCs were seeded at a density of 5 × 10^4^ cells/well. After 24 h of culture, the Ti samples with attached cells were gently washed with PBS, fixed in 2.5% glutaraldehyde, dehydrated in a graded ethanol series and freeze-dried. After sputter coating with carbon, the cell morphology was observed by FE-SEM.

### In vitro osteogenesis of MSCs

The expression of osteogenisis-related genes was evaluated using the real-time PCR. The cells were seeded with 5 ×10^4^ cells/well and cultured for 48 h. Then the medium was changed into osteogenic medium containing 10 mM β-glycerophosphate, 50 μg/ml ascorbic acid and 10^−7^ M dexamethasone. After further culture of 7 and 14 days, the total RNA was isolated using the TRIzol reagent. Then 2 μg RNA from each sample was reversed transcribed into complementary DNA (cDNA) using the PrimeScript RT reagent kit. The expression of osteogenesis-related genes including collagen type I (COL1), runt-related transcription factor 2 (RUNX2), alkaline phosphatase (ALP), bone morphogenetic protein-2 (BMP-2), osterix (OSX) and osteocalcin (OCN) was quantified using real-time PCR. The PCR reaction was carried out using SYBR Premix Ex Taq™ II on the CFX96™ real-time PCR System. The relative expression levels for each gene of interest were normalized to that of the housekeeping gene GAPDH. The PCR primers were synthesized as shown in Table 1.

**Table 1:** Primers used for real-time PCR

Gene	Forward primer sequence (5′-3′)	Reverse primer sequence (5′-3′)
ALP	AACGTGGCCAAGAACATCATCA	TGTCCATCTCCAGCCGTGTC
BMP-2	CAACACCGTGCTCAGCTTCC	TTCCCACTCATTTCTGAAAGTTCC
COL1	GCCTCCCAGAACATCACCTA	GCAGGGACTTCTTGAGGTTG
OCN	GGTGCAGACCTAGCAGACACCA	AGGTAGCGCCGGAGTCTATTCA
RUNX2	CCATAACGGTCTTCACAAATCCT	TCTGTCTGTGCCTTCTTGGTTC
OSX	AAGGCAGTTGGCAATAGTGG	TGAATGGGCTTCTTCCTCAG
GAPDH	GGCACAGTCAAGGCTGAGAATG	ATGGTGGTGAAGACGCCAGTA

#### ALP staining

The cell inoculation and culture was the same as in the real-time PCR assay. After culturing for 7 and 14 days, the cells on the Ti samples were washed with PBS and fixed with 4% paraformaldehyde. ALP was stained with the BCIP/NBT ALP color development kit for 15 min. The samples were washed thoroughly with PBS to acquire the images.

#### Collagen secretion

The cell inoculation and culture was the same as in the real-time PCR assay. After culture of 7 and 14 days, the cultures were washed by PBS and fixed by 4% paraformaldehyde. Then the collagen secretion was stained by 0.1wt% Sirius red in saturated picric acid for 18 h. The unbound stain was removed in 0.1 M acetic acid and then the images were collected. To quantitatively assess the collagen secretion, the stain on the samples was eluted in 500 μl destain solution (0.2 M NaOH/methanol 1:1) and the optical density at 540 nm was measured using a spectrophotometer.

#### ECM mineralized nodule displaying

The cell inoculation and culture was the same as in the real-time PCR assay. After culturing for 14 and 28 days, the cells were washed twice with PBS and then fixed with 60% isopropanol for 1 min. After rehydrating with distilled water for 2–3 min, the ECM mineralized nodules formed by MSC culture were stained with 1wt% alizarin red for 3 min. After thorough washing with distilled water, the images were taken.

### In vivo osseointegration

#### Implant surgery

The animal experiment was approved by the Animal Research Committee of the Fourth Military Medical University and conducted in accordance with the international standards on animal welfare. Twenty female Sprague–Dawley rats, 3 months old with a weight of approximately 250 g, were randomly divided into 5 groups: antimiR-138 group, antimiR-control group, CS group, MAO group and polished Ti (PT) group (n = 8 for each group). The femur model was applied for implantation in this study, and the procedure of the surgery was similar to the literature procedure [41]. Briefly, after anaesthesia by pelltobarbitalum natricum, the hind limbs were prepared by shaving and cleaning with ethanol and 10% povidone iodine. Then, an incision was made over the distal side of the knee. Gentle dissection was used to move the ligament and patella aside to expose the intercondylar notch of the distal femur. For placement of the screw implant, dental burs and a surgical motor (OsseoSet 200, Nobel Biocare AB, Sweden) with a low rotational drill speed (800 rpm) were used to generate a 2.8 mm cylindrical hole at the intercondylar notch of the femur parallel to the long axis of the bone cooled continuously with sterile saline solution. Implants were inserted into the femoral medullary canal, followed by incision suture. The animals received an injection of gentamicin (1 mg/kg) immediately after surgery and for 5 days after the surgery.

#### Micro-CT evaluation

Specimens (femurs containing implants) were extracted (n = 4 per group) and fixed in 4% paraformaldehyde for 24 h. Then, they were scanned by a micro-CT scanner (Y.Cheetah, YXLON International GmbH, Germany). The region of interest (ROI) was defined as the region at 2 mm height from 0.5 µm below the growth plate and 200 µm around the implant surface, and the images were analysed via VGStudio Max 2.2 (Volume Graphic, Germany). The bone volume per total volume (BV/TV), the mean trabecular thickness (Tb.Th), the mean trabecular number (Tb.N) and the mean trabecular separation (Tb.Sp) were assessed within the ROI zone.

#### Van Gieson staining

After micro-CT scanning, the specimens were dehydrated with graded alcohol and embedded in methyl methacrylate. Afterwards, thin sections (approximately 50 µm in thickness) parallel to the long axis of the implants were prepared using a macrocutting and grinding system (SP1600 and SP2600, Leica, Germany). Sections were then polished and stained with 1.2% trinitrophenol and 1% acid fuchsin (Van Gieson staining). Finally, a qualitative analysis of the bone formation was performed with a standard light microscope (M80, Leica, Germany) equipped with a digital image analysis system (Image-Pro Plus software, USA). The bone-to-implant contact (BIC) was also determined.

#### Line-scanning of the bone-to-implant interface

Line scanning by energy-dispersive X-ray spectroscopy (EDX, Hitachi, Japan) was applied for a more detailed analysis of the bone-to-implant interface. After having been fixed with 4% paraformaldehyde, dehydrated with graded alcohol, and embedded in methyl methacrylate, the bone-to-implant interfaces of the embedded samples were scanned by FE-SEM. The line profiles of C, O, Ca, P, and Ti elements were analysed from the implant side to the medullary cavity side by EDX.

### Statistical analysis

The one-way ANOVA and Turkey post hoc tests were used to determine the level of statistical significance of difference among groups. *p* < 0.05, 0.01 and 0.001 was set to be significant, highly significant and extremely significant, respectively.

## Results

### Characterization of the CS-antimiR-138/HA PEM-functionalized microporous Ti surface

#### Contact angle measurement

**Fig. 1:**
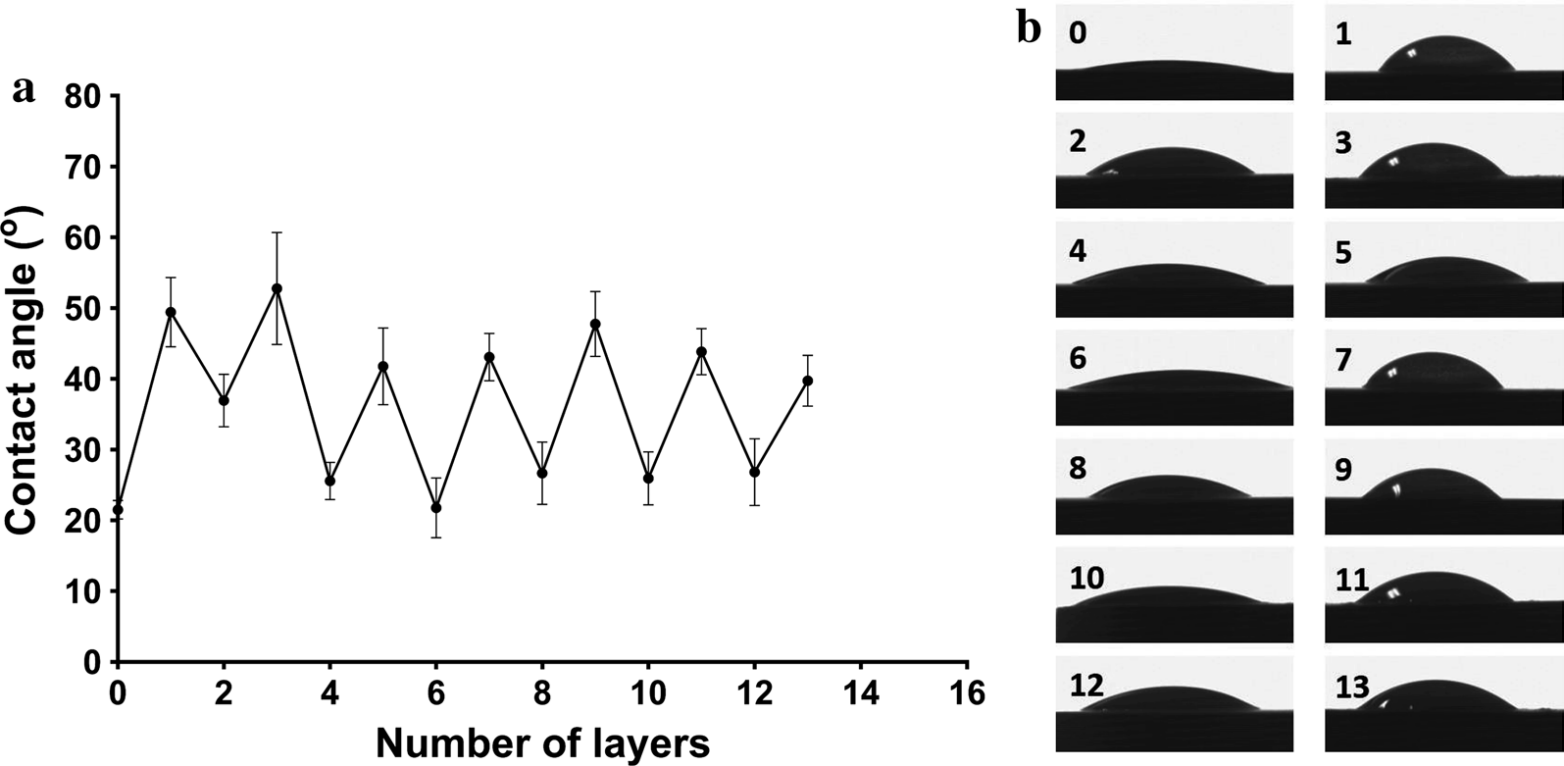
Water contact angles (**a**) and the lateral views of water drops (**b**) on the MAO surface after LbL processes

During the growth of the CS-antimiR-138/HA PEM-functionalized microporous Ti surface, we monitored the wettability of each layer. As shown in Fig. 1, the naked MAO Ti surface (0 layer) was hydrophilic with the lowest water contact angle of ∼ 20°. The initially adsorbed APTES and GA layers, named layers 1 and 2, respectively, were both less hydrophilic than the naked MAO, showing contact angles of approximately 50° and 35°. After the growth of layer 3 on the surface, which was CS, the contact angle decreased. The following layers displayed an alternating decrease or increase in their contact angles until layer 13. In general, the HA layer on the surface produced lower contact angles than the CS-antimiR-138 layer.

#### SEM and fluorescent observation

**Fig. 2:**
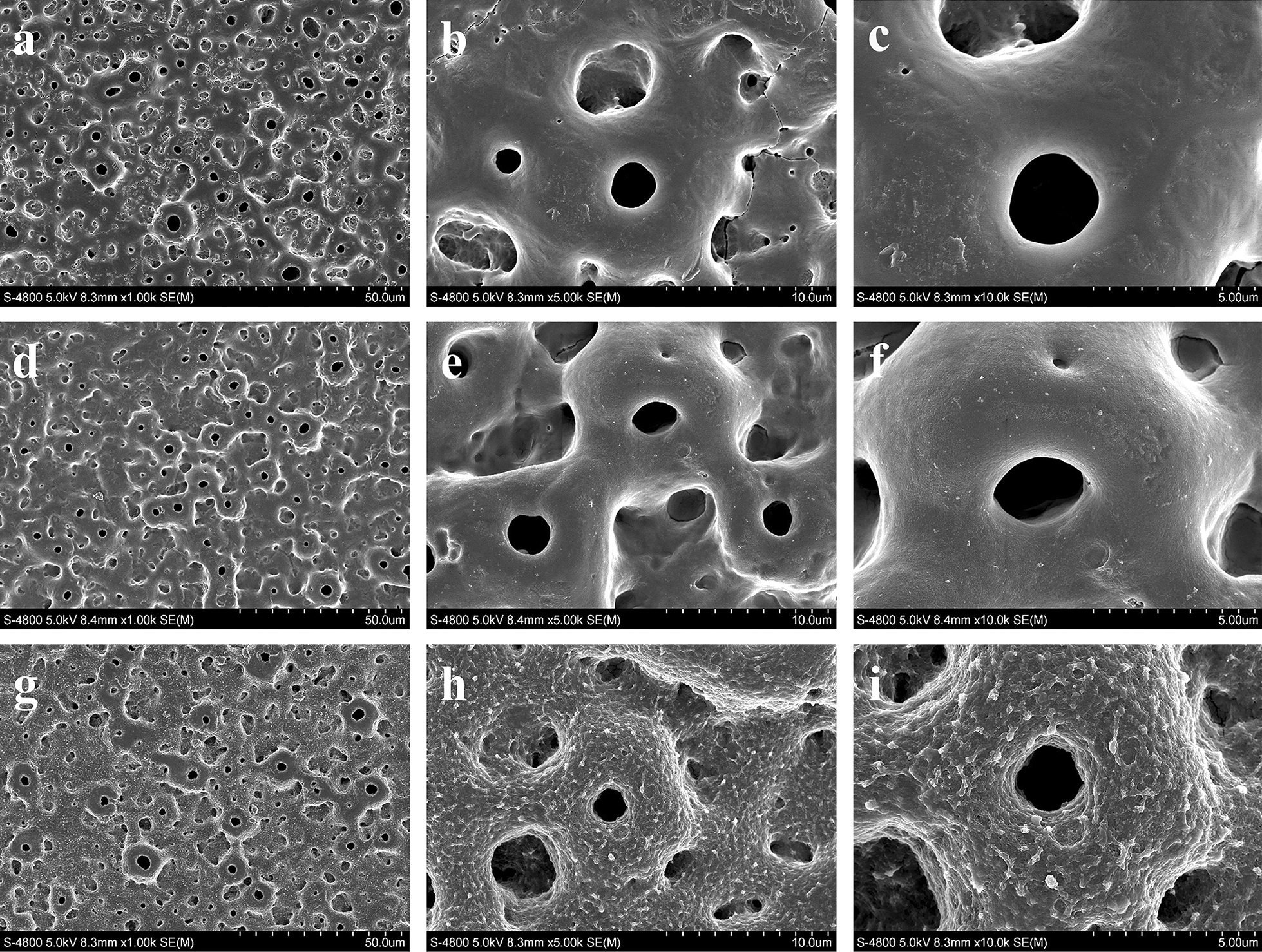
Morphology of the MAO Ti surfaces before and after CS-miRNA/HA PEM-functionalized inspected by FE-SEM: (**a**, **b** and **c**) pictures of different magnification for the naked MAO surface, (**d**, **e** and **f**) pictures of silane glutaraldehyde functionalized MAO surface, (**g**, **h** and **i**) pictures of 5 layers for the CS-miRNA/HA PEM-functionalized MAO surface

The morphology of the MAO Ti surfaces before and after PEM functionalization was observed by FE-SEM (Fig. 2). The naked MAO surface exhibited the typical MAO microporous structure (Fig. 2a–c). After the silane-glutaraldehyde coupling process, there was no apparent change in the structure (Fig. 2d–f). For the CS-miRNA/HA PEM-functionalized microporous Ti surface, the CS-miRNA NPs were distributed evenly across the microporous structure (Fig. 2g–i). The presence of the miRNA NPs slightly decreased the diameters of the micropores, and the NPs even completely filled some of the smaller micropores by attaching to their sidewalls.

**Fig. 3:**
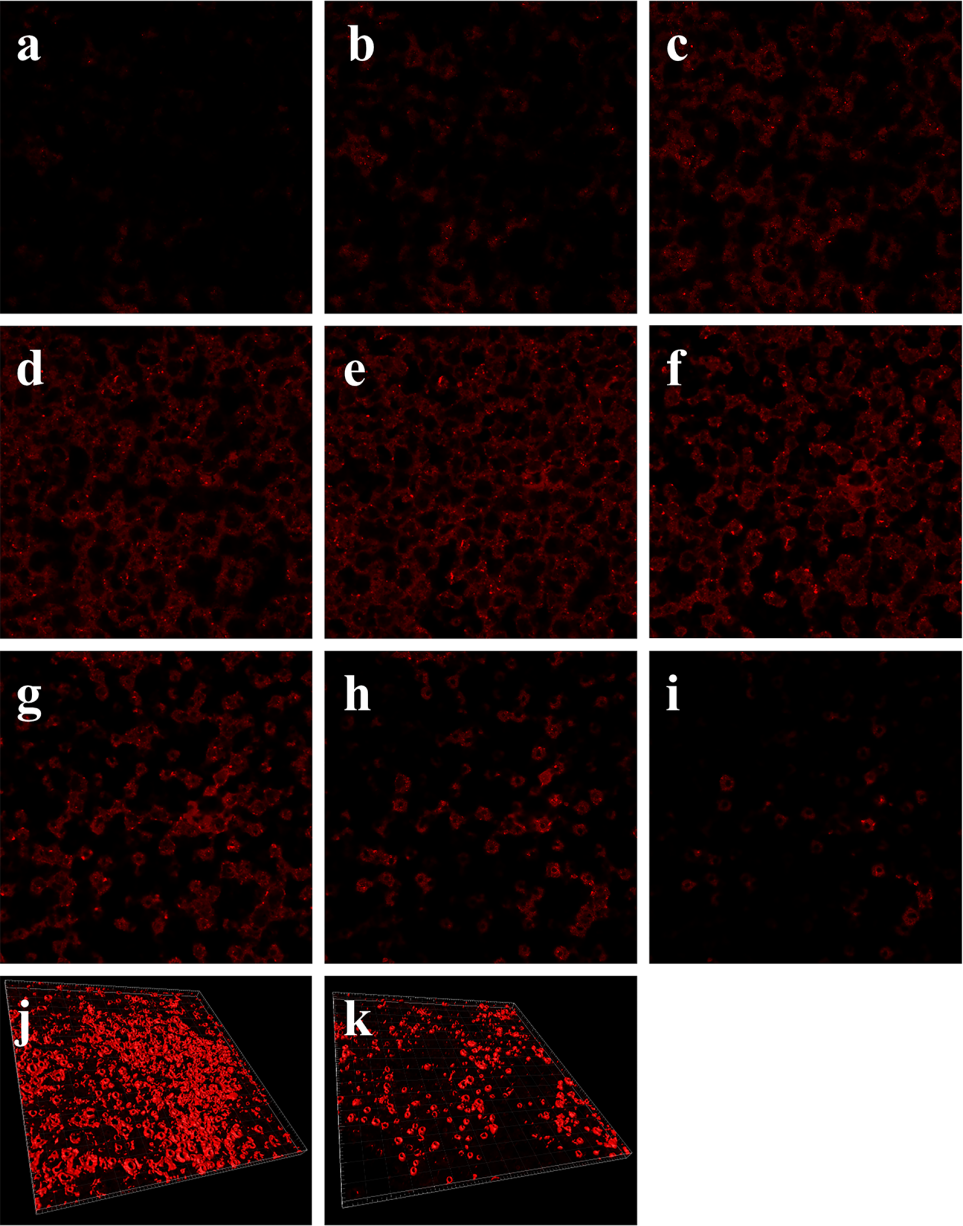
Fluorescence confocal laser scanning microscope of CS-miRNA/HA PEM-functionalized MAO surface with Cy3-labeled miRNAs: **a** the top layer starting to display fluorescence and **b**–**i** the continuing layers from top to down with an interlayer distance of 400 nm. The fluorescence 3-D images of Cy3-labeled miRNAs on the MAO surfaces before (**j**) and after (**k**) 7 days of incubation in cell culture medium at 37 °C

To observe the miRNA loading and release, the Cy3-labelled miRNAs were used to fabricate the CS-antimiR-138/HA PEM-functionalized microporous Ti surface. The surface was observed by laser scanning confocal microscopy from the surface to the bottom with a layer-by-layer scanning approach with an interlayer distance of 400 nm (Fig. 3a–i). From the fluorescence images, the miRNAs were distributed relatively evenly and internalized into the pores. Figure 3a and i show the top and bottom layers of the CS-antimiR-138/HA PEM coating, respectively. The total thickness of the CS-antimiR-138/HA PEM coating was estimated to be approximately 3.2 µm. From the middle layers (Fig. 3b–h), we can observe many fluorescence rings attributed to the CS-miRNA NPs that attached to the sidewalls of the micropores. To display the miRNA release, the three-dimensional images (Fig. 3j, k) were reconstructed through the software after layer-by-layer scanning, and they show the miRNA amount before and after 7 days of immersion in the culture medium. A certain amount of miRNAs was retained on the surface and the sidewalls of the micropores after 7 days, indicating a high Ti surface retention of the CS-miRNA NPs and potential sustained release of the miRNAs from the surface.

### Quantification of miRNA loading and release of the CS-miRNA/HA PEM-functionalized microporous Ti surface

**Fig. 4:**
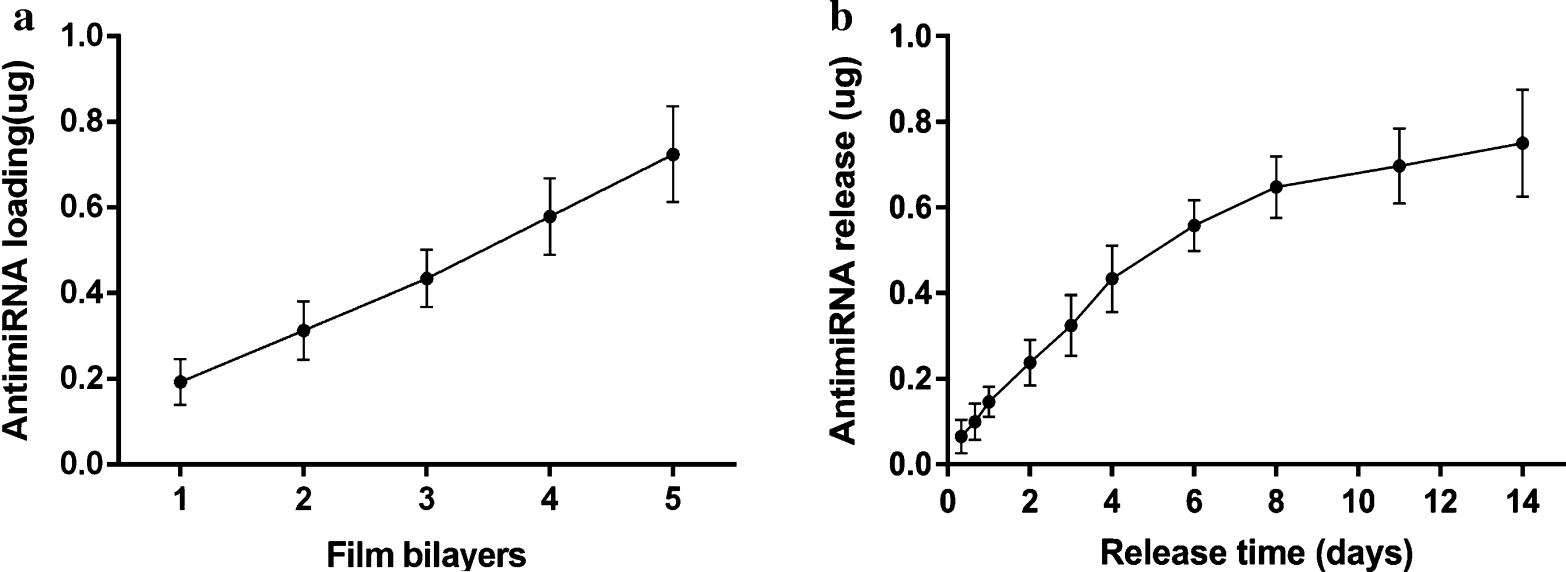
Quantifcation of antimiR-138 on novel CS-antimiR-138/HA PEM-functionalized MAO surface. **a** Accumulated antimiR-138 loading amount with increased number of biofunctional layers, **b** accumulated antimiR-138 release profile from the coated surface

The accumulative loading profiles were calculated (Fig. 4). The accumulated miRNA loading increased gradually with the layer number, indicating that the LbL approach can be used to control the miRNA loading (Fig. 4a). The miRNAs released steadily from the CS-miRNA/HA PEM-functionalized microporous Ti surface without apparent initial burst release (Fig. 4b). In the first 4 days, nearly 50% of the loaded miRNAs were released from the functionalized surface. After 14 days, a total of ~ 0.8 μg was released, which was nearly 100% of the loaded amount.

### Transfection efficiency of the CS-miRNA/HA PEM-functionalized microporous Ti surface

**Fig. 5:**
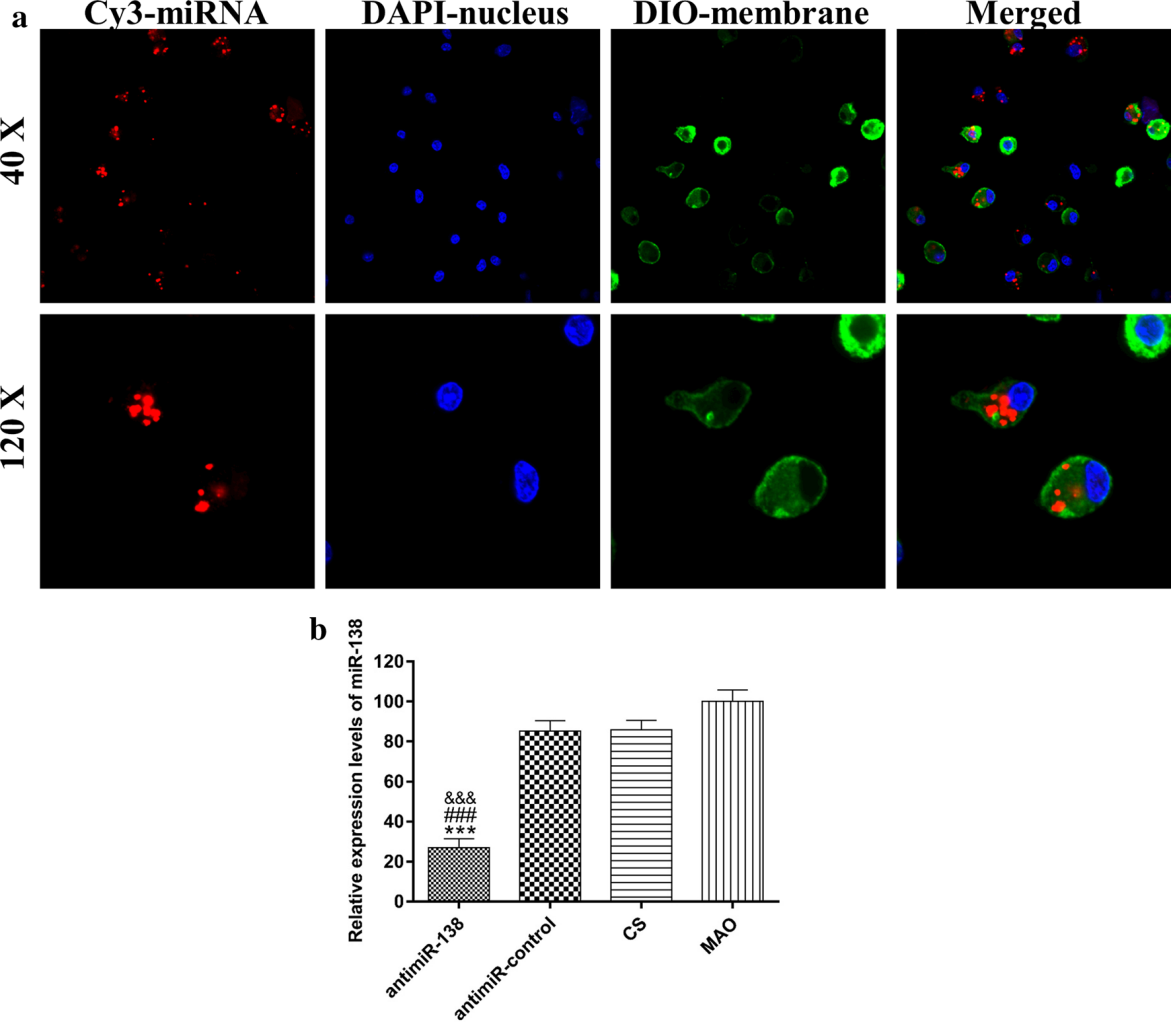
**a** Fluorescence images of 40× (upper) and 120× (lower) showing the uptake of Cy3-labelled miRNAs (red) by cells after 24 h of culture on the CS-miRNA/HA PEM functionalized MAO surface: Cell nucleus was visualized using DAPI (blue) and membrane using DIO (green). **b** Downregulation of microRNA expression by 5 layers miRNA coating. ***p < 0.001 vs the CS-antimiR-control/HA PEM-functionalized MAO surface; ^###^*p* < 0.001 vs the CS-coated MAO surface; ^$$$^*p* < 0.001 vs the naked MAO surface

To observe the internalization of the miRNAs into the cells, Cy3-labelled antimiR-138 was used to fabricate the functionalized Ti surface before seeding MSCs on the surface for 24 h of transfection. The miRNAs were red, and the cell nucleus and membrane were stained with blue and green colours, respectively (Fig. 5a). We observed that after 24 h of incubation, the miRNAs were mainly located in the cell body and around the nucleus, suggesting successful uptake of the CS-miRNA NPs by the cells. Most cells have intracellular miRNAs, indicating a high transfection efficiency of the functionalized Ti surface.

To assess the downregulatory effect of the CS-antimiR-138/HA PEM-functionalized microporous Ti surface on the level of intracellular miR-138, the amount of intracellular miR-138 was measured by miRNA PCR (Fig. 5b). The miR-138 level in MSCs seeded on the CS-antimiR-138/HA PEM-functionalized microporous Ti surface was successfully downregulated by approximately 70%.

### Cell attachment and morphology

**Fig. 6:**
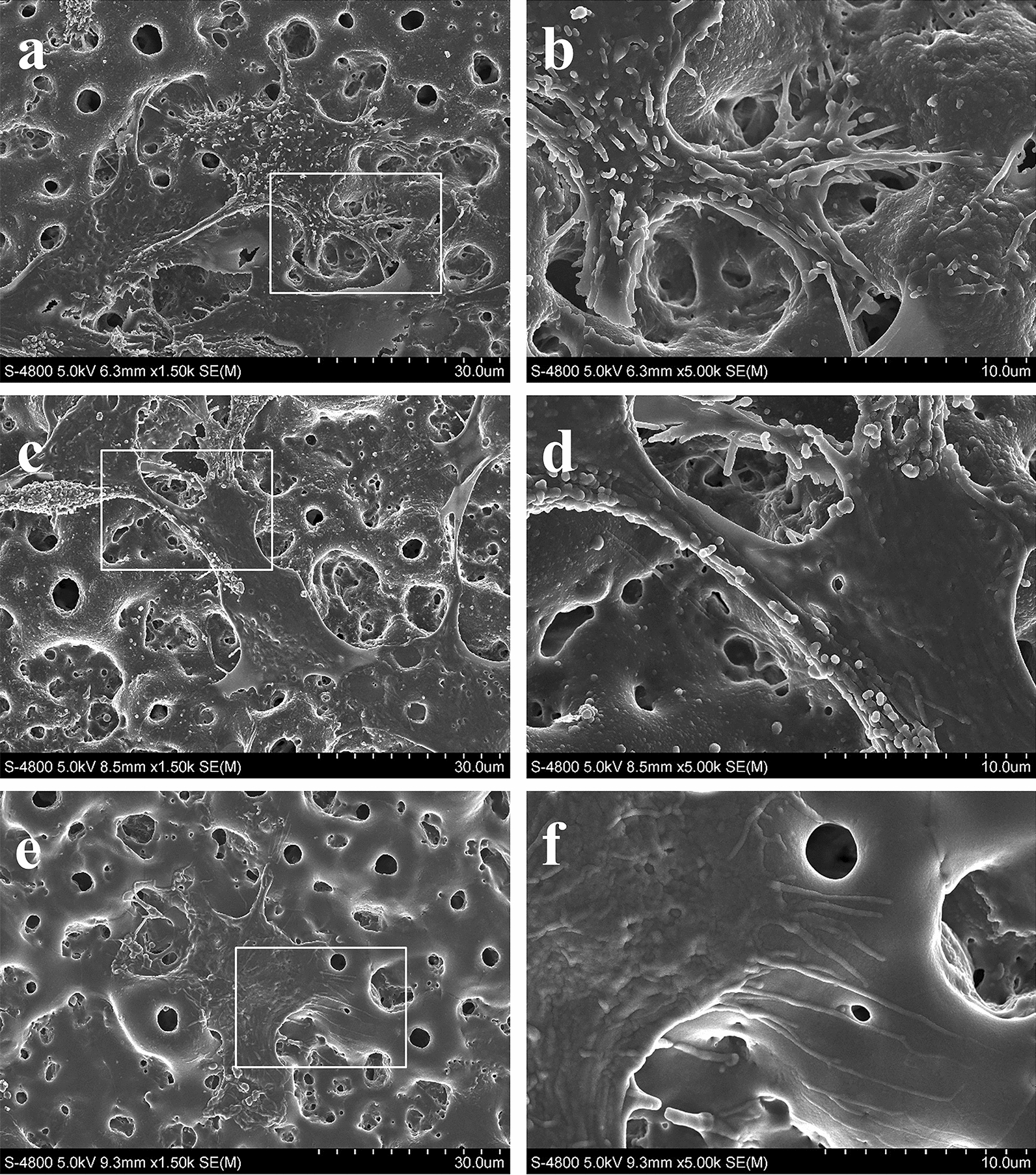
FE-SEM pictures showing the cell morphology after 24 h of incubation on different samples: **a**, **b** the CS-antimiR-138/HA PEM-functionalized MAO surface (**c**, **d**) the antimiR-control/HA PEM-functionalized MAO surface and (**e**, **f**) the naked MAO surface

The MSC attachment and cell spreading on the Ti samples were observed by FE-SEM (Fig. 6). Generally, the cells attached well to the microporous structure, and showed very similar polygonal cell morphology and a similar spreading area on the microporous Ti surface and the PEM-functionalized microporous Ti surfaces with antimiR-138 or antimiR-control. However, on the CS-antimiR-138/HA PEM-functionalized microporous Ti surface, the filopodia extended from the cells seemed to be thicker than those on the other two surfaces. The cellular filopodia on the CS-antimiR-138/HA PEM-functionalized microporous Ti surface were anchored in the micropores. This phenomenon was not observed on the other two control surfaces. Furthermore, there were many granules on the dorsal side of the cells on the CS-antimiR-138/HA PEM-functionalized microporous Ti surface, indicating that the cells on the CS-antimiR-138/HA PEM-functionalized microporous Ti surface have a more mature secretory phenotype.

### Effect of the CS-antimiR-138/HA PEM-functionalized microporous Ti surface on osteogenesis in vitro

**Fig. 7:**
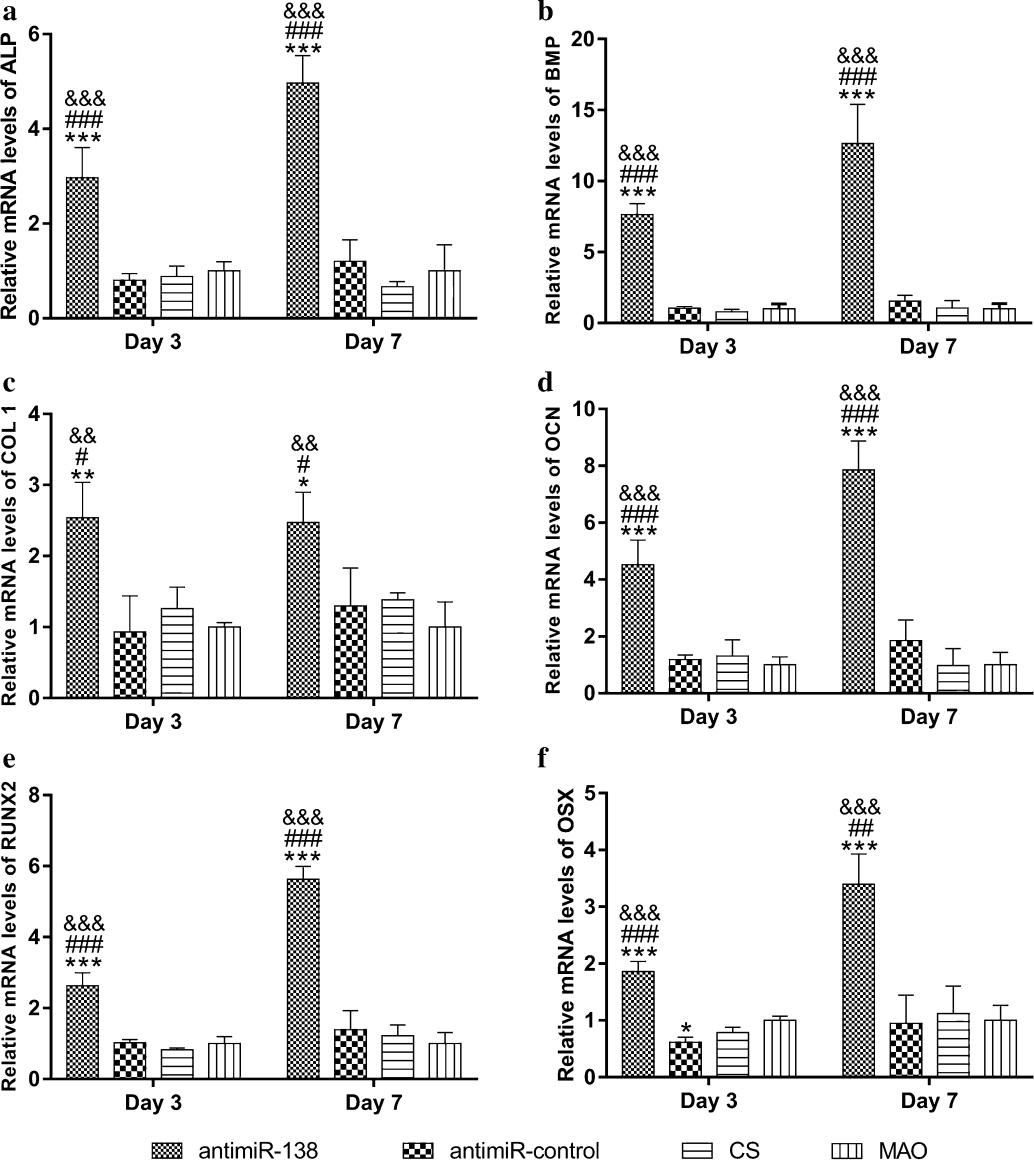
Relative expression of **a** ALP, **b** BMP, **c** Col1, **d** OCN, **e** OSX and **f** RUNX2 by MSCs cultured on different samples. After culturing in the growth medium for 24 h, the medium was changed to osteogenic medium for further culture of 3 and 7 days. All values are normalized to GAPDH. *, **, ***p < 0.05, 0.01 and 0.001 vs the naked MAO surface; ^#,##,###^*p* < 0.05, 0.01 and 0.001 vs the CS-coated MAO surface; ^@@, @@@^*p* < 0.01 and 0.001 vs the CS-antimiR-control/HA PEM-functionalized MAO surface

The expression levels of osteogenesis-related genes were assessed by real-time PCR (Fig. 7). Generally, the CS-antimiR-138/HA PEM-functionalized microporous Ti surface induced strikingly higher expression levels of BMP-2, OCN, COL1, ALP, OSX and RUNX2 by MSCs than the CS-antimiR-control/HA PEM-functionalized microporous Ti surface, the CS-coated surface and the naked MAO surface. There was no obvious difference in the expression levels of those genes among the CS-antimiR-control/HA PEM-functionalized microporous Ti surface, the CS-coated surface or the naked MAO surface.

**Fig. 8:**
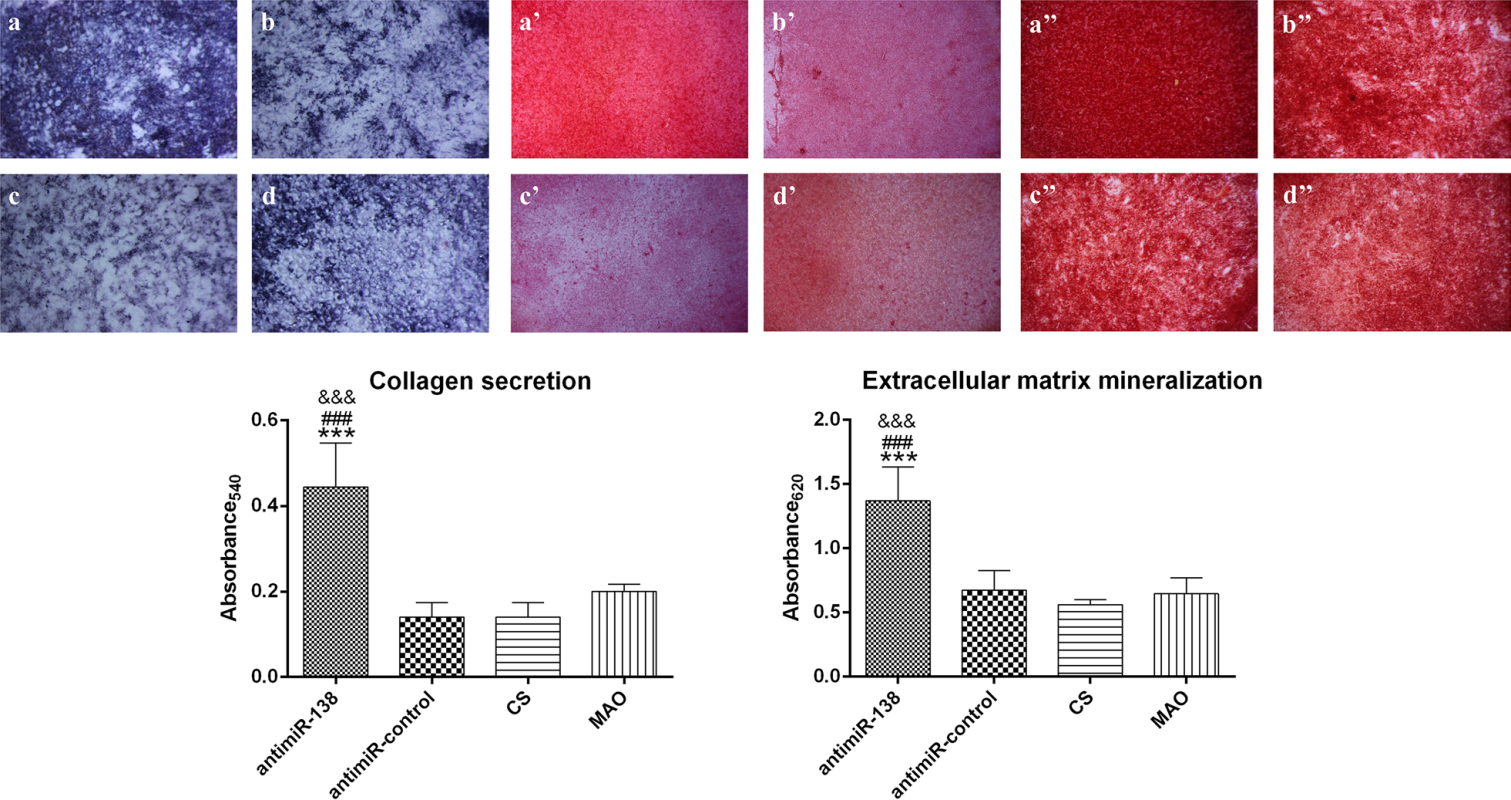
The ALP product and collagen secretion after 7 days of culture as well as the ECM mineralization after 14 days of culture: (**a**, **a’** and **a’’**) the CS-antimiR-138/HA PEM-functionalized MAO surface, (**b**, **b’** and **b’’**) the CS-antimiR-control/HA PEM-functionalized MAO surface, (**c**, **c’** and **c’’**) the CS-coated MAO surface and (**d**, **d’** and **d’’**) the naked MAO surface. The bottom panel shows the semi-quantitative results. ***p < 0.001 vs the naked MAO surface; ^###^*p* < 0.001 vs CS-coated MAO surface; ^&&&^*p* < 0.001 vs the CS-antimiR-control/HA PEM-functionalized MAO surface

Then, the ALP production and collagen secretion after 7 days of culture as well as the ECM mineralization after 14 days of culture were assessed (Fig. 8). The MSCs produced the most ALP on the CS-antimiR-138/HA PEM-functionalized microporous Ti surface after 7 days of osteogenic induction (Fig. 8a–d). There was no obvious difference in the induced ALP production levels among the antimiR-control/HA PEM-functionalized microporous Ti surface, the CS-coated surface or the naked MAO surface.

Collagen secretion was stained with Sirius red, and the results are shown in Fig. 8a′–d′. The optical images show that much more collagen was secreted by the cells on the CS-antimiR-138/HA PEM-functionalized microporous Ti surface compared to the collagen secretion on the antimiR-control/HA PEM-functionalized microporous Ti surface, the CS-coated surface and the naked MAO surface. The quantitative results indicate that the amount of secreted collagen on the CS-antimiR-138/HA PEM-functionalized microporous Ti surface was approximately double that on the naked MAO surface and triple that on the antimiR-control/HA PEM-functionalized microporous Ti surface and the CS-coated surface.

Next, the ECM mineralized nodules were stained via alizarin red, and the results are shown in Fig. 8a″–d″. The mineralized nodules formed by the MSCs were observed in the optical images after 14 days of culture on all four surfaces, and the CS-antimiR-138/HA PEM-functionalized microporous Ti surface generated the most mineralized nodules. The quantitative results reveal that the CS-antimiR-138/HA PEM-functionalized microporous Ti surface induced the formation of approximately twice as many mineralized nodules than the three control groups did.

### In vivo osseointegration of the CS-antimiR-138/HA PEM-functionalized microporous Ti implant

**Fig. 9:**
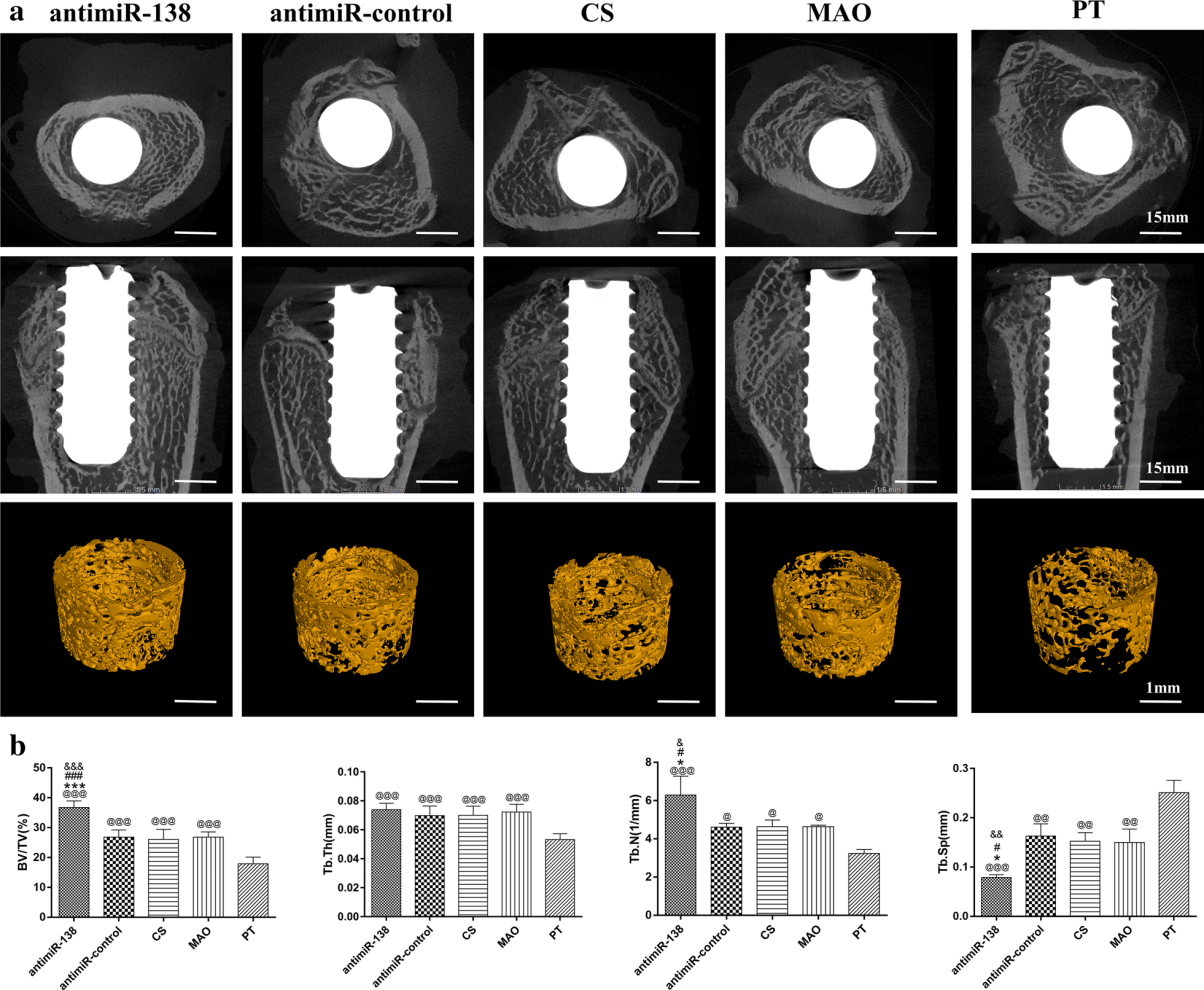
**a** Transverse and vertical 2-D images and 3-D reconstructed views (ROI, 200 µm from the implant surface) of the Micro-CT analysis to show the new bone formation around the Ti implants at 4 weeks. Scale bar: 15 mm on the 2-D images and 1 mm on the 3-D ones. **b** The bone volume per total volume (BV/TV), the mean trabecular thickness (Tb.Th), the mean trabecular number (Tb.N) and the mean trabecular separation (Tb.Sp) within the ROI zone. ^@, @@, @@@^*p* < 0.05, 0.01 and 0.001 vs the PT surface *, ***p < 0.05 and 0.001 vs the naked MAO surface; ^#,###^*p* < 0.05, and 0.001 vs the CS-coated MAO surface; ^&,&&,&&&^*p* < 0.05, 0.01 and 0.001 vs the CS-antimiR-control/HA PEM-functionalized MAO surface

Micro-CT analysis was performed to evaluate new bone formation and osseointegration in vivo (Fig. 9). The reconstructed 3D stereoscopic images of the new bone formation (yellow) around the implants are shown in Fig. 9a. The quantitative evaluation of the bone-volume ratio (BV/TV) and trabecular architecture are shown in Fig. 9b. The analysis of BV/TV, Tb.Th, Tb.N and Tb.Sp in the ROI revealed that the bone trabecula around the PT surface was the thinnest and sparsest. Compared to the three MAO control groups, the CS-antimiR-138/HA PEM-functionalized microporous Ti surface induced noticeably more new bone formation (with BV/TV and Tb.N increased by approximately 40% and Tb.Sp decreased by approximately half (*p* < 0.05)).

**Fig. 10:**
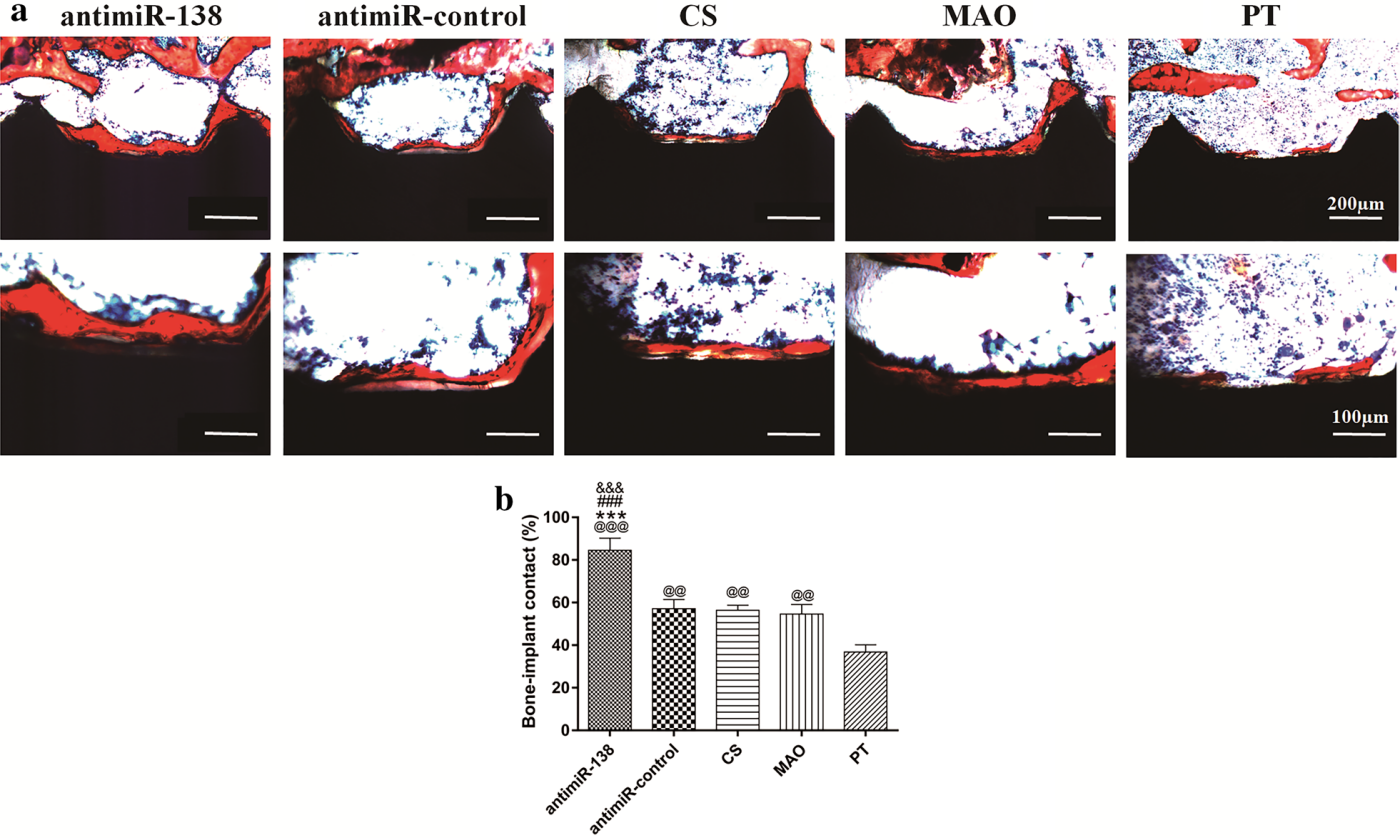
**a** Histological images of undecalcified sections of new bone formation around the implants at 4 weeks by Van Gieson staining. The bone tissue was stained in red color. Scale bar: 200 µm on the above and 100 µm on the below. **b** Histomorphometric measurement of the BIC in the ROI. ^@@,@@@^*p* < 0.01 and 0.001 vs the PT surface ***p < 0.001 vs the naked MAO surface; ^###^*p* < 0.001 vs the CS-coated MAO surface; ^&&&^*p* < 0.001 vs CS-antimiR-control/HA PEM-functionalized MAO surface

To evaluate the tissue healing progress around the implants, we performed a histological analysis of the bone-to-implant interface by Van Gieson staining. New bone (red) was formed around the implants in all groups 4 weeks after implantation (Fig. 10a). Compared to the PT implant with thin and discontinuous new bone formation, the CS-antimiR-control/HA PEM-functionalized microporous Ti implant, the CS-coated implant and the naked MAO implant induced more new bone formation. The CS-antimiR-138/HA PEM-functionalized microporous Ti implant generated the largest bone volume and the best bone continuity around the implant surface. Quantitative measurement of the BIC (Fig. 10b) shows a similar trend. The BIC increased from approximately 38% on the PT implant to approximately 85% on the CS-antimiR-138/HA PEM-functionalized microporous Ti implant.

**Fig. 11:**
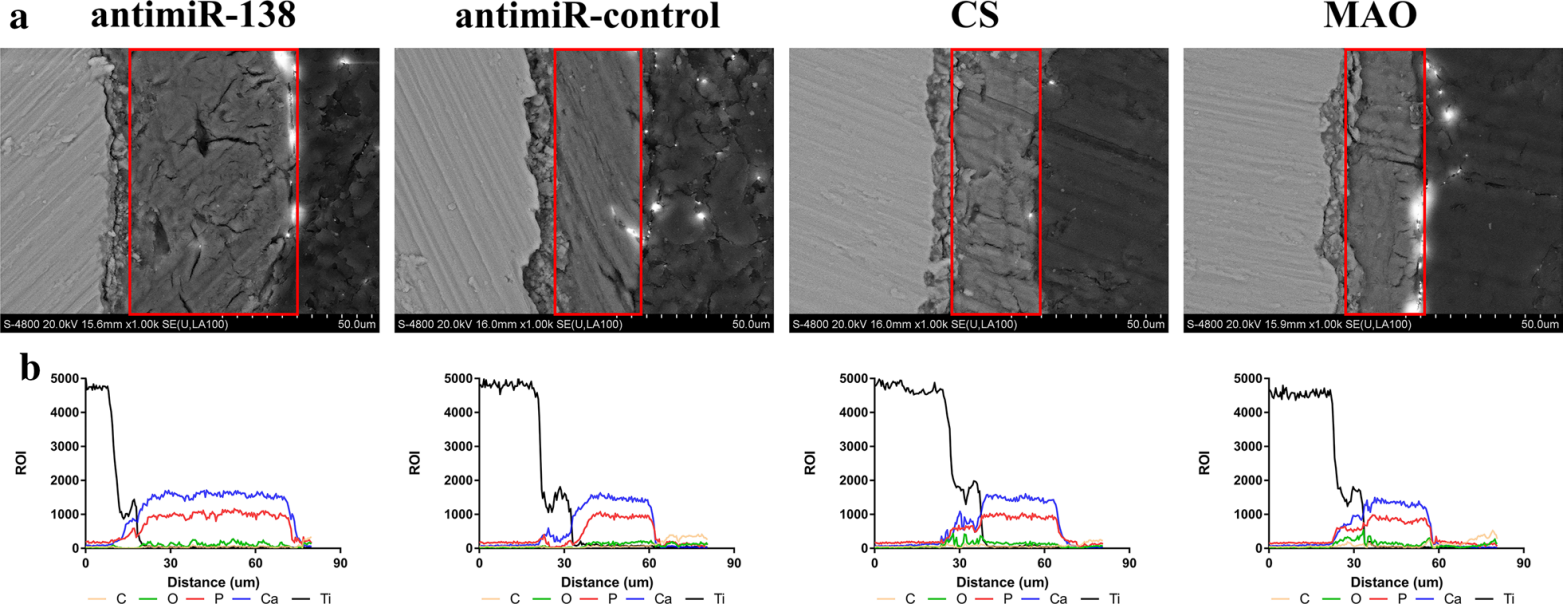
**a** FE-SEM pictures showing the new bone on the bone-to-implant interface of different samples. **b** EDX line scanning of the elements in the direction perpendicular to the bone-to-implant interface. Red frame indicates the new bone area on the implant surface

The cross-section of the bone-to-implant interface was inspected by FE-SEM (Fig. 11a). The distribution of elements including C, O, P, Ca, and Ti across the interface was analysed by EDX line scanning (Fig. 11b). The thickness of new bone with Ca and P was measured. The antimiR-138 PEM-functionalized microporous Ti surface induced the thickest new bone. The thickness of the new bone was approximately 18, 25, 27, 27, and 50 μm for the PT implant, the naked MAO implant, the CS-coated MAO implant, the antimiR-control PEM-functionalized microporous Ti implant and the CS-antimiR-138/HA PEM-functionalized microporous Ti implant, respectively.

## Discussion

The development of implants with quicker and tighter osseointegration is important to satisfy clinical requirements. Loading the implant surface with therapeutic oligonucleotides is a promising approach for accelerated tissue healing/integration [15, 42–44]. Song et al. created a siRNA biofunctionalized implant with a titanium nanotube array coating by cathodic electrodeposition of a CS-siRNA complex [45]. Previously, we conducted proof-of-concept studies to fabricate antimiR-138 lipoplex-functionalized Ti implants via lyophilization [22], which demonstrated the feasibility of the miRNA functionalization of Ti implants. However, a more biocompatible carrier instead of liposomes should be used to avoid cytotoxicity, and a sustainable release should be obtained by controlling the loading and delivery kinetics of antimiR-138.

In this study, multilayers of CS and HA fabricated by the LbL self-assembly technique were used to load miRNA on the Ti implant surface to realize a larger miRNA loading amount and a long-lasting release. This technique is based on the alternate adsorption of polyanions and polycations via electrostatic interactions on a charged substrate and has been widely used for the controlled release of biological molecules [46–48]. To firmly attach the LbL films to the MAO Ti surface, the silane reaction is utilized, as the rigid covalent bond provides a firm chemically crosslinked connection between the MAO surface and chitosan. UV irradiation produces abundant hydroxyl groups on the MAO surface, which can be reacted with the ethoxy groups of APTES [37]. The terminal amine group can then be reacted with the aldehyde groups of glutaraldehyde, which is commonly applied for crosslinking with biomolecules [49, 50]. Then, chitosan can be covalently conjugated to the MAO Ti surface via the APTES-GA layer, which guaranteed the stability of the self-assembled multilayers on the Ti surface. Furthermore, due to its hydrophobicity and positive charge, chitosan was used as the superficial layer to promote protein adsorption and cell adhesion [51]. The FE-SEM images reveal that the MAO treatment generated a microporous structure topography with potential higher loading capacity [22]. The following silane reaction and PEM deposition formed a homogeneous surface coating without affecting the original MAO microtopography. Using laser scanning confocal microscopy, it was shown that the CS-miRNA complexes indeed entered deeply into the micropores and adhered to the inner sidewalls. The contact angle results again demonstrated that the self-assembled multilayers of chitosan and HA were successfully introduced onto the MAO Ti surface [52, 53].

The miRNA loading profile was analysed by fluorescence intensity measurement, which is one of the most sensitive methods for RNA quantification [54]. The cumulative loading amount of miRNA increased linearly with the number of layers. The in vitro miRNA release profile exhibited a phase of linear and fast release in the first 4 days, followed by a slower release phase. The observed long-lasting release kinetics can be attributed to the electrostatic interaction between the negatively and positively charged polymers in the PEM [30, 55, 56]. Furthermore, the MAO micropores can protect the nanoparticles from premature release due to an increased contact area and reduced shear force, contributing to the long-lasting release. Taken together, a long-lasting miRNA release of over 2 weeks was observed from the PEM-functionalized MAO Ti surface.

Then, the miRNA cellular uptake and the transfection efficiency of the CS-antimiR-138/HA PEM-functionalized microporous Ti surface were assessed. After cell plating on CS-antimiR-138/HA PEM-functionalized microporous Ti surfaces and transfection for 24 h, abundant miRNAs were taken up by the cells, indicating a high transfection efficiency. This was further verified by miRNA-PCR. The intracellular target miRNA level was successfully downregulated by approximately 70%. The high transfection efficiency was expected to be related to the microporous Ti surface and the miRNAs retained in the micropores, which leads to a localized concentration of miRNA. The direct cellular contact to this localized availability of nucleic acids results in improved transfection efficiency [57–59]. This effect is known as substrate-mediated gene delivery [57, 60]. In addition, it has been reported that stability of CS/pDNA or CS/RNA complexes would not change in storage under 4 °C [61, 62]. Here, the CS-antimiR-138/HA PEM-functionalized microporous Ti surface show consistent transfection efficiency over 14 days when stored at 4 °C (Additional file 1: Fig. S1 and 5b), indicating good storability for transportation and use.

High cytocompatibility without cytotoxicity or impairment of cell functions is an elementary requirement for the clinical application of biofunctionalized implants. Our data demonstrate that the CS-antimiR-138/HA PEM-functionalized microporous Ti surface satisfies this requirement (Additional file 1: Fig. S2). This is attributed to the biocompatibility of CS and HA and APTES/GA functional groups in the PEM. Nolte et al. found very low toxicity of HA/CS coatings [63]. Kuddannaya et al. found that poly(dimethylsiloxane) surfaces functionalized with APTES + GA + proteins showed enhanced MSC osteogenic induction with no cytotoxicity, which was related to the APTES/GA functional groups [49, 64]. SU-8 surfaces functionalized with collagen type I or fibronectin via covalent conjugation through APTES + GA were biocompatible and used for long-term cell culture [65]. The cell attachment and spread influence the ensuing cell functions, and were observed by FE-SEM. The cells exhibited a very similar cell spreading behaviour with abundant cell lamellipodia stretching out on all surfaces, which further illustrates the high cytocompatibility of the CS-antimiR-138/HA PEM-functionalized microporous Ti surface. The CS-antimiR-138/HA PEM-functionalized MAO Ti surface induced thicker filopodia, which were anchored in the micropores and may lead to a more rigid osseointegration. A more mature secretory cell phenotype was observed on the CS-antimiR-138/HA PEM-functionalized MAO Ti surface, which is likely due to the inhibition of the intracellular miR-138 function by antimiR-138 to the promote osteogenic differentiation of MSCs [49].

The use of a proper miRNA is pivotal for achieving satisfactory osteogenic activity for the PEM-functionalized implant surface. miR-138 is an important regulator in the development of the osteoblast phenotype [18, 22, 66]. It has been reported that miR-138 inhibits osteogenic differentiation of MSCs in vitro and the formation of bone in vivo. By inhibiting the focal adhesion kinase signalling pathway, antimiR-138 can promote the expression of osteoblast-specific genes, ALP activity, and ECM mineralization of MSCs, and enhance in vivo ectopic bone formation, suggesting that antimiR-138 is a good therapeutic molecule for enhancing osteogenesis [18]. Yan et al. also confirmed that antimiR-138 delivery increased the phosphorylation of ERK1/2, and subsequently increased the osteogenesis-related genes in antimiR-138-transfected MSCs [67]. Tsukamoto et al. found that the inhibition of miR-138 with an LNA-modified anti-miR-138 oligonucleotide enhanced the osteogenic differentiation of MSCs in vitro [68]. In addition, our previous data also demonstrated the efficiency of antimiR-138 in promoting osteogenic differentiation in vitro [22]. Therefore, the osteogenesis-inducing capacity of the antimiR-138-functionalized microporous coatings in vitro and in vivo was assessed. Excitingly, antimiR-138 resulted in enhanced osteogenic activity in vitro and excellent bone formation in vivo, which is consistent with the previous reports.

## Conclusions

In this study, we developed a novel CS-antimiR-138/HA PEM-functionalized microporous Ti implant, which was prepared via silane-glutaraldehyde coupling to activate the MAO Ti surface and facilitate the chemical conjugation with biolayers composed of HA and chitosan using an LbL technique. The coating exhibited sustained release of CS-antimiR-138, which was taken up efficiently by the cells and caused significant knockdown of the target miR-138 without inducing apparent cytotoxicity. Ti implants functionalized with antimiR-138 markedly enhanced the osteogenic differentiation of MSCs and the osseointegration in an in vivo rat model, thus being a very promising method to expedite the clinical implant osseointegration. This functionalization may thus represent a valuable tool for biomedical applications where a controlled, long-lasting delivery is needed and provide meaningful insights regarding miRNA loading into other biomaterials.

## Supplementary information

**Additional file 1: Figure S1.** Storable stability of CS-antimiR-138/HA PEM-functionalized microporous Ti samples. After storage for different durations (7 and 14 days), the transfection efficiency was measured to assess the stability during storage. **Figure S2.** (a) Cell viability measured by CCK-8 at 24 h after transfection and (b) LDH amount released by cells during the first 24 h after transfection.

## Data Availability

All data generated or analyzed during this study are included in this published article.
