# Supplementary material for: Chitosan-miRNA functionalized microporous titanium oxide surfaces via a layer-by-layer approach with a sustained release profile for enhanced osteogenic activity
Source: J Nanobiotechnology. 2020 Sep 9;18:127. doi: 10.1186/s12951-020-00674-7 (PMC7487814; doi:10.1186/s12951-020-00674-7)
Supplement: Supplementary file 1 — Additional file 1: Figure S1. Storable stability of CS-antimiR-138/HA PEM-functionalized microporous Ti samples. After storage for different durations (7 and 14 days), the transfection efficiency was measured to assess the stability during storage. Figure S2. (a) Cell viability measured by CCK-8 at 24 h after transfection and (b) LDH amount released by cells during the first 24 h after transfection. [file 12951_2020_674_MOESM1_ESM.docx]

**Additional file**

**Chitosan-miRNA Functionalized Microporous Titanium Oxide Surfaces via a Layer-by-layer Approach with a Sustained Release Profile for Enhanced Osteogenic Activity**

Kaimin Wu,^1†^ Mengyuan Liu, ^2†^ Nan Li, ^3†^ Li Zhang^4^, Fanhui Meng^4^,

Lingzhou Zhao^5*^, Min Liu^1*^ and Yumei Zhang^4*^

^1^Department of Stomatology, Navy 971st Hospital, Qingdao 266071, China

^2^Oral Research Center, Qingdao Municipal Hospital, Qingdao 266071, China

^3^Third department of cadre’s ward, Navy 971st Hospital, Qingdao 266071, China

^4^State Key Laboratory of Military Stomatology & National Clinical Research Center for Oral Diseases & Shaanxi Key Laboratory of Stomatology, Department of Prosthodontics, School of Stomatology, The Fourth Military Medical University, Xi’an 710032, China

^5^State Key Laboratory of Military Stomatology & National Clinical Research Center for Oral Diseases & Shaanxi Engineering Research Center for Dental Materials and Advanced Manufacture, Department of Periodontology, School of Stomatology, The Fourth Military Medical University, Xi’an 710032, China

^†^ These authors contribute equally to this work.

*Corresponding authors:

Department of Periodontology and Oral Medicine, School of Stomatology, The Fourth Military Medical University, No. 145 West Changle Road, Xi’an 710032, China. Dr. Lingzhou Zhao; Tel.: +86-29-84776093; E-mail: [zhaolingzhou1983@hotmail.com](mailto:zhaolingzhou1983@hotmail.com)

Department of Stomatology, Navy 971st Hospital, No. 22 Minjiang Road, Qingdao 266071, China. Dr. Min Liu; Tel.: +86-532-51870130; E-mail: [dentist401@163.com](mailto:dentist401@163.com)

Department of Prosthetic Dentistry, School of Stomatology, The Fourth Military Medical University, No. 145 West Changle Road, Xi’an 710032, China. Prof. Yumei Zhang; Tel.: +86-29-84776329; E-mail: [wqtzym@fmmu.edu.cn](mailto:wqtzym@fmmu.edu.cn)

**Storage stability of CS-antimiR-138/HA PEM-functionalized microporous Ti samples**

After fabrication, the Ti samples were stored at 4°C for a total period of up to 14 days. Mesenchymal stem cell transfection was performed on the CS-antimiR-138/HA PEM-functionalized Ti implant, the intracellular miR-138 amount was measured as described above. High stability with no obvious decrease in transfection efficiency was observed when samples were stored at 4°C (Fig. S1).


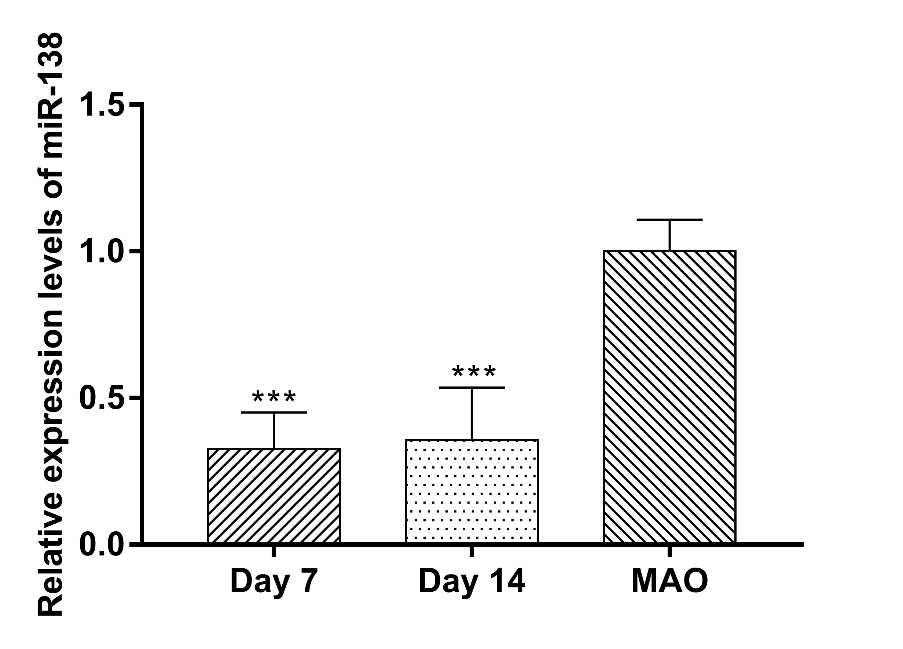


**Fig. S1** Storable stability of CS-antimiR-138/HA PEM-functionalized microporous Ti samples. After storage for different durations (7 and 14 days), the transfection efficiency was measured to assess the stability during storage. *** *p* < 0.001 vs the naked MAO surface.

**Cell viability and Lactate dehydrogenase activity assay**

The CCK-8 assay was employed to evaluate the cell viability according to the manufacturer’s instruction. Briefly, the reaction medium was formed by mixing serum free ɑ-MEM and CCK-8 at a ratio of nine to one. MSCs of 2.5×10^4^/cm^2^ were inoculated on the Ti samples placed in the 24 well plates. After 24 h of culture, the medium was removed and the Ti samples were washed with PBS twice. Then 400 μl reaction medium was added to each well and incubated at 37°C for 3 h. The supernatant was transferred to a 96-well plate and the optical density (OD) was determined using a spectrophotometer (Bio-tek) at 450 nm wavelength. The LDH activity in the culture medium was used as an index of cytotoxicity. After 24 h of culture, the culture medium was collected and centrifuged, and the supernatant was used for the LDH activity assay. The LDH activity was determined spectrophotometrically according to the manufacturer’s instruction.

Cell viability and LDH results are shown in Fig. S2a, b. The cells cultured on the tissue culture plate (blank control), the naked MAO surface, the CS-coated MAO surface, and the PEM-functionalized microporous Ti surfaces with antimiR-138 or antimiR-control showed similar cell viability and LDH, indicating that the CS-antimiR-138/HA PEM-functionalized microporous Ti surface possesses good cytocompatibility and shows no cytotoxicity.


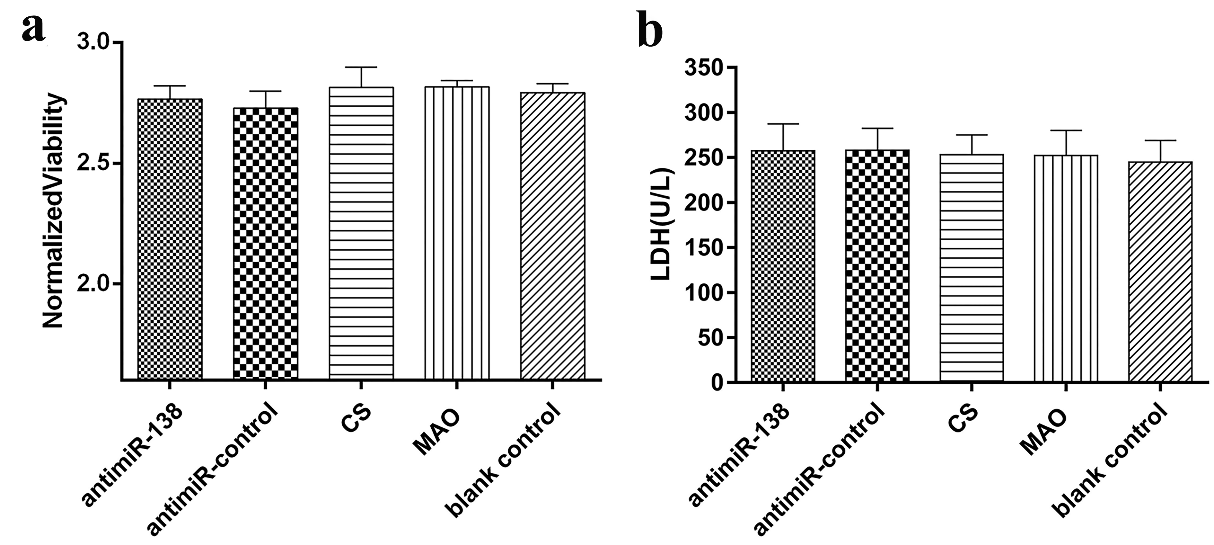


**Fig. S2** (**a**) Cell viability measured by CCK-8 at 24 h after transfection and (**b**) LDH amount released by cells during the first 24 h after transfection.
